# Increasing Mechanical Properties of 2-D-Structured Electrospun Nylon 6 Non-Woven Fiber Mats

**DOI:** 10.3390/ma9040270

**Published:** 2016-04-07

**Authors:** Chunhui Xiang, Margaret W. Frey

**Affiliations:** 1 Department of Apparel, Events and Hospitality Management, Iowa State University, Ames, IA 50011, USA; 2 Department of Fiber Science and Apparel Design, Cornell University, Ithaca, NY 14853, USA; mfw24@cornell.edu

**Keywords:** increasing, electrospun, nylon 6, mechanical properties, 2-D structure, solvent bonding, annealing, solvent vapor exposure, non-woven fiber mats

## Abstract

Tensile strength, Young’s modulus, and toughness of electrospun nylon 6 non-woven fiber mats were improved by increasing individual nanofiber strength and fiber–fiber load sharing. Single-walled carbon nanotubes (CNTs) were used as reinforcement to increase the strength of the electrospun nylon 6 nanofibers. Young’s modulus, tensile strength, and toughness of the nylon 6 non-woven fiber mats electrospun from 20 wt % solutions increased 51%, 87%, and 136%, respectively, after incorporating 1 wt % CNTs into the nylon 6 nanofibers. Three methods were investigated to enhance fiber–fiber load sharing: increasing friction between fibers, thermal bonding, and solvent bonding. The addition of beaded nylon 6 nanofibers into the non-woven fiber mats to increase fiber-fiber friction resulted in a statistically significantly increase in Young’s modulus over comparable smooth non-woven fiber mats. After annealing, tensile strength, elongation, and toughness of the nylon 6 non-woven fiber mats electrospun from 20 wt % + 10 wt % solutions increased 26%, 28%, and 68% compared to those from 20 wt % solutions. Solvent bonding with formic acid vapor at room temperature for 30 min caused increases of 56%, 67%, and 39% in the Young’s modulus, tensile strength, and toughness of non-woven fiber mats, respectively. The increases attributed to increased individual nanofiber strength and solvent bonding synergistically resulted in the improvement of Young’s modulus of the electrospun nylon 6 non-woven fiber mats.

## 1. Introduction

Electrospinning can produce polymer fibers with diameters in the range of nanometers to a few micrometers. The electrospun nanofibers have many potential applications such as optical materials [1], sensor materials [2], nanocomposite materials [3], tissue scaffolds [4], wound dressing [5], drug delivery systems [6], filtration [7], and protective clothing [8]. However, because electrospun fiber mats have very poor mechanical properties due to the random orientation within the fibers and broad distribution of fiber diameter, their actual uses have been limited. Hence, an enhancement in mechanical properties of the electrospun fiber mats is very important from an industrial point of view [9].

Mechanisms of deformation in non-woven fabrics are based on fiber and bond deformations [10]. The inherent strength of fibers produced by electrospinning is dependent on polymer type, crystallization rate, and degree of crystallinity. Nylon 6 has a rapid crystallization rate and has been shown to produce strong electrospun fibers [11]. Due to this property, nylon 6 is often used in industry as a coating for filter media [12]. Park *et al.* [9] suggested the combined effects of the higher degree alignment, the surface nanocoating, and the formation of internal networks of polyelectrolytes on nylon-6 fibers resulted in higher tensile strength. Papkov *et al.* [13] has demonstrated improvements in modulus and strength of individual electrospun polymer nanofibers with a reduction in their fiber diameter. They reported that reduction of fiber diameter resulted in simultaneous increases in elastic modulus, true strength, and toughness.

Kim *et al.* [14] reported that the electrospun thermoplastic polyurethane elastomer (TPU) fiber mats showed nonlinear elastic and inelastic characteristics, which might be due to slippage of crossed fiber (nonbonded or physical bonded structure) and breakage of the electrospun fibers at junctions (point-bonded or chemical bonding structure) and demonstrated that the point-bonded structures of fiber mats played an important role in the load-bearing component as determined in loading-unloading component tests. 

Theoretical and experimental studies have shown that carbon nanotubes (CNTs) have an extremely high Young’s modulus, similar to that of the in-plane value for graphite (~1000 GPa) [15]. CNTs are considered to be the ideal reinforcing agent for high-strength polymer composites, because of their high mechanical strength, electrical conductivity, and thermal conductivity. Good interfacial adhesion between the CNTs and the polymer matrix is essential for efficient load transfer in the composite [16]. CNTs are often used as a reinforcing phase to improve mechanical properties of fibers by: (a) acting as a physical reinforcement; and (b) acting as a nucleation agent to increase overall crystallinity of fibers. Therefore, CNT-reinforced polymer composites have potential applications in defense and aerospace applications, where high-strength and light-weight components are of primary importance. Many polymers are presently being investigated as host matrices for CNTs, and the resulting composites have been found to show improved mechanical properties. A direct mixing of multiwalled carbon nanotubes (MWNTs) and polystyrene led to a 36%–42% increase in elastic stiffness and a 25% increase in tensile strength with the incorporation of only 1 wt % of MWNTs into the polystyrene matrix [17]. Mahfuz *et al.* [18] reported that tensile tests on single nylon 6 filmaments had demonstrated that Young’s modulus and strength of the nanophased filaments had increased by 220% and 164% with the addition of 1 wt % of MWNTS. In addition to the strength of individual fibers, the number of crossings per nanofiber, intersections per unit area, total nanofiber crossings in the mat, and three-dimensional joint morphology all play an important role in the mechanical properties of non-woven nanofiber mats [19]. Nylon 6 is of significantly industrial importance because of its excellent strength, toughness, and wearing resistance [11,15].

In the present study, single-walled carbon nanotubes (CNTs) were incorporated into the nylon 6 nanofibers during electrospinning to increase the individual fiber strength. Beaded electrospun nylon 6 fibers were introduced to uniform nylon 6 nanofiber non-woven fiber mats to increase fiber-fiber bonding and hence to improve the mechanical properties of 2-D-structured electrospun nylon 6 non-woven fiber mats. The as-spun nylon 6 non-woven fiber mats were also exposed over formic acid vapor at room temperature to increase fiber–fiber bonding.

## 2. Results and Discussion

### 2.1. Increase Nylon 6 Nanofiber Strength

**Figure 1:**
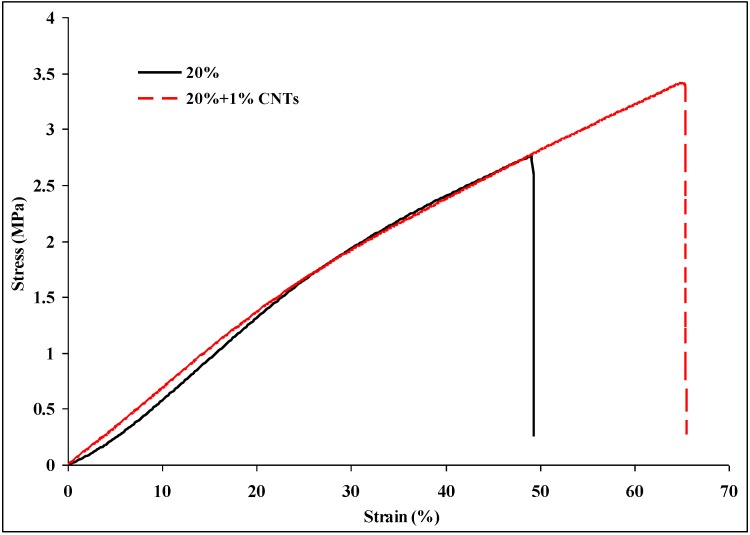
Typical stress-strain plots of nylon 6 non-woven fiber mats electrospun from 20 wt % nylon 6 solutions with and without 1 wt % carbon nanotubes (CNTs) suspensions.

Figure 1 shows the typical stress-strain plots of nylon 6 non-woven fiber mats electrospun from 20 wt % nylon 6 in 88% formic acid solutions with and without 1 wt % (based on the weight of nylon 6) CNTs. Table 1 is the summary of the tensile properties of nylon 6 non-woven fiber mats electrospun from 20 wt % nylon 6 in 88% formic acid solutions with and without 1 wt % CNTs. Young’s modulus, tensile strength, elongation, and toughness (calculated by integrating the area under the stress-strain curves) of the nylon 6 non-woven fiber mats increased 51%, 87%, 18%, and 136%, respectively, after incorporating 1 wt % CNTs into the nylon 6 nanofibers. Therefore, the composite electrospun nylon 6/CNTs non-woven fiber mats have a higher stiffness, strength, and ductility. The enhancement in the mechanical properties might originate from the good dispersion of CNTs as well as the strong interaction between CNTs and nanofibers. Good interfacial adhesion between the CNTs and the nylon 6 nanofiber is essential for efficient load transfer in the non-woven fiber mats [16]. The improvement of the strength property by incorporating CNTs into the nanofibers was expected and consistent with the reported results in the literature. Bazbouz *et al.* [19] reported that the tensile strength of the nylon 6 nanofiber mats electrospun from 20 wt % containing only 1.0 wt % MWCNTs (Multiwall carbon nanotubes) was increased by 25%, compared with the nylon 6 mats. They also reported that the strain at break (elongation) of the nylon 6/MWCNTs nanofiber mats was decreased by 18%. However, the elongation of the electrospun nylon 6/SWNTs non-woven fabrics increased 18%. Jeong *et al.* [20] reported the elongation of electrospun polyvinyl alcohol (PVA) non-woven fabrics increased by incorporating MWNTs into composite nanofibers when the weight percentage of filler (MWNTs) was lower than 3 wt %. The reason for this is that, at a low filler wt %, the load transfer to the inner nanotubes is low, which enhances the tensile properties. The initial increase in the tensile strength and tensile modulus (*i.e.*, for the composites containing 1 wt % and 2.5 wt % of filler) is attributed to the high degree of orientation of the filler nanotubes in the wrapped nanofibers.

### 2.2. Increase Fiber-Fiber Bonding

#### 2.2.1. Beaded Fiber Effect

**Figure 2:**
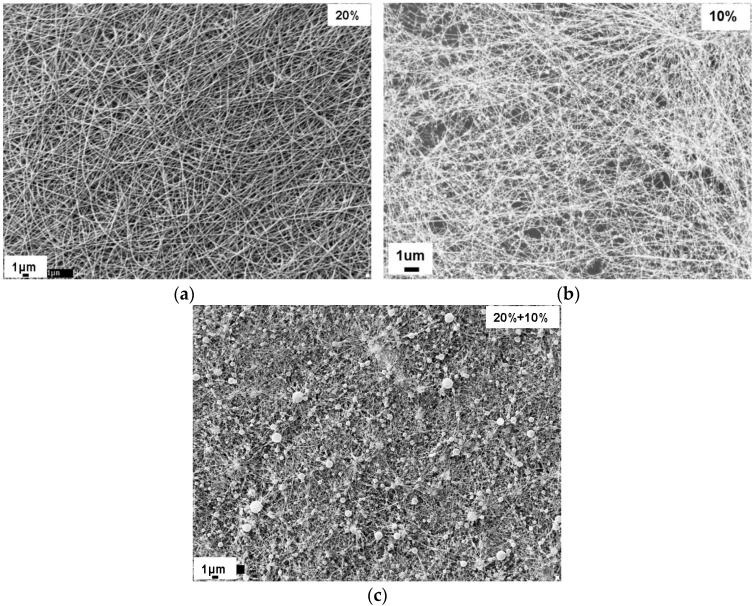
SEM imaging of nylon 6 nanofibers electrospun from (**a**) 20 wt % nylon 6; (**b**) 10 wt % nylon 6; and (**c**) 20 wt % and 10 wt % in 88% formic acid solutions.

**Figure 3:**
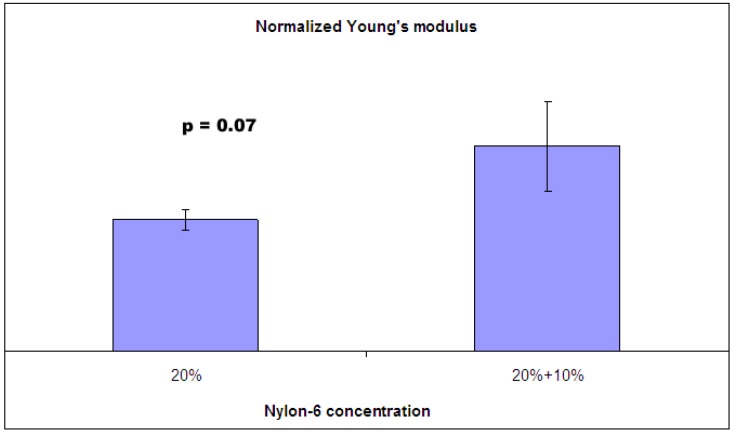
Beads effect on Young’s modulus of electrospun nylon 6 non-woven fiber mats.

In order to improve the mechanical stability of the electrospun nanofibrous mat, the interfiber bonding could be introduced by controlling the electrospinning process or by post-processing. If a spinning solvent has relatively low volatility (a high boiling point), a considerable amount of solvent remains within nanofibers deposited on target, and wet fibers are then formed. The presence of residual solvent in the electrospun nanofiber facilitates the bonding of intersecting fibers [21]. Figure 2 shows the morphology of nylon 6 non-woven fiber mats electrospun from 20 wt %, 10 wt %, and 20 wt % paralleled with 10 wt % solutions. Nylon 6 nanofibers electrospun from 20 wt % solutions (Figure 2a) have uniform fiber diameters, while nylon 6 nanofibers electrospun from 10 wt % solutions (Figure 2b) shows a highly beaded structure. Therefore, the non-woven fiber mats produced by paralleled electrospinning 20 wt % and 10 wt % solutions resulted in a combination of uniform fibers and beaded fibers (Figure 2c). With the addition of the beaded nylon 6 nanofibers into the non-woven fiber mats, the nylon 6 non-woven fiber mats electrospun from 20 wt % and 10 wt % demonstrated a statistically significant increase in Young’s modulus when compared with nylon 6 non-woven fiber mats electrospun from 20 wt % (Figure 3). Formic acid has a boiling point of 101 °C, which is relatively low volatility. The addition of beaded nylon 6 nanofibers into the comparable smooth non-woven fiber mats resulted in the increase of fiber–fiber friction.

#### 2.2.2. Annealing Nylon 6 Non-Woven Fiber Mats

**Figure 4:**
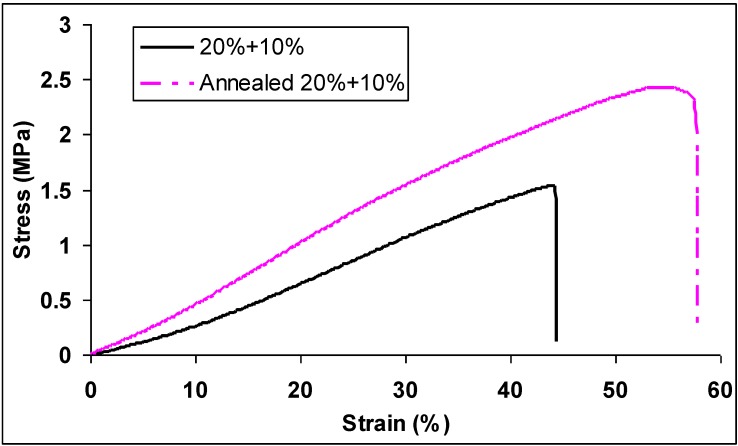
Typical stress-strain plots of nylon 6 non-woven fiber mats electrospun from 20 wt % and 10 wt % solutions before and after annealing at 70 °C for 24 h.

Figure 4 shows the typical stress-strain plots of nylon 6 non-woven fiber mats electrospun from 20 wt % and 10 wt % solutions. After annealing, tensile strength, elongation, and toughness of the nylon 6 non-woven fiber mats electrospun from 20 wt % and 10 wt % solutions increased 26%, 28%, and 68% compared with those from 20 wt % solutions. Young’s modulus, tensile strength, elongation, and toughness of nylon 6 non-woven fiber mats electrospun from 20 wt % and 10 wt % solutions before and after annealing are summarized in Table 2. For random-laid non-woven fiber mats, once the web is formed, the fibers must be bonded together to stabilize the web and allow it to exhibit the desired mechanical properties. Both individual fiber mechanical properties and cohesion between fibers can be improved by free and constrained annealing of the electrospun non-woven fiber mats at temperatures slightly greater than the base polymer’s glass transition temperature. The annealing process reduced internal stresses (decrease shrinkage) and increased crystallinity and crystalline alignment within fibers. Choi *et al.* [22] reported that the physical properties of the electrospun poly(etherimide) (PEI) fiber web were improved by thermal treatment above its glass transition temperature.

#### 2.2.3. Solvent Bonding Effect

**Figure 5:**
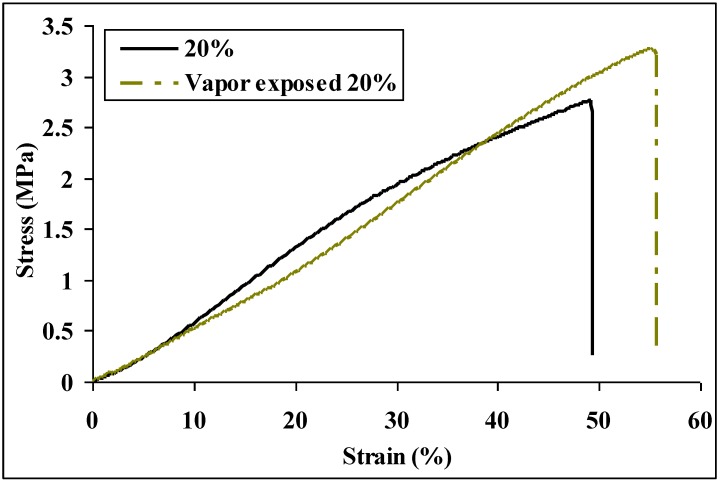
Typical stress-strain plots of nylon 6 non-woven fiber mats electrospun from 20 wt % solutions before and after formic acid vapor exposure for 30 min at room temperature.

**Figure 6:**
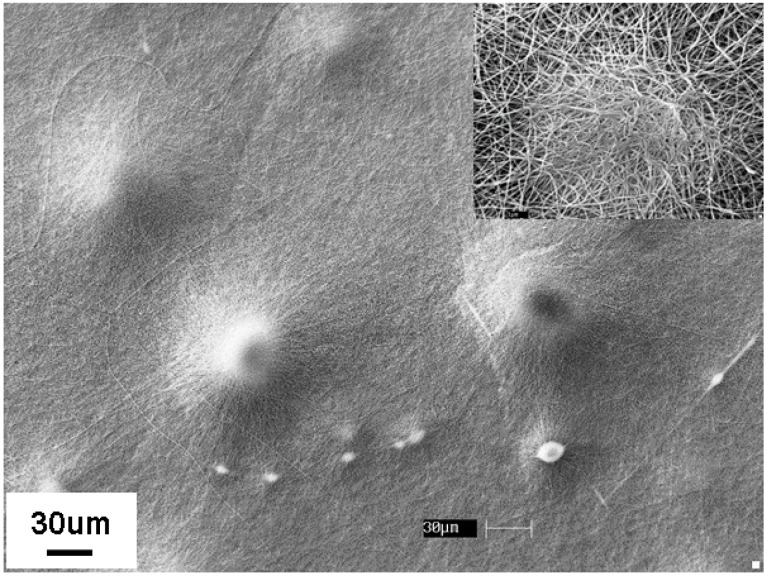
SEM imaging of nylon 6 non-woven fiber mats electrospun from 20 wt % solutions after formic acid vapor exposure for 30 min at room temperature.

Figure 5 shows the typical stress-strain plots of nylon 6 non-woven fiber mats electrospun from 20 wt % solutions before and after 30 min formic acid vapor exposure at room temperature. Young’s modulus, tensile strength, and toughness of the nylon 6 non-woven fiber mats increased 56%, 67%, and 39%, respectively, after formic acid vapor exposure for 30 min at room temperature. The tensile properties are summarized in Table 3. SEM images in Figure 6 show evidence that the fiber-fiber bonding increased after vapor exposure. Hence, the mechanical properties improved. Distinct bonding points are formed where droplets of formic acid vapor have condensed on the non-woven fabric surface. The higher magnification image of one bonded region shows clear evidence of fusion points between overlapping fibers.

**Figure 7:**
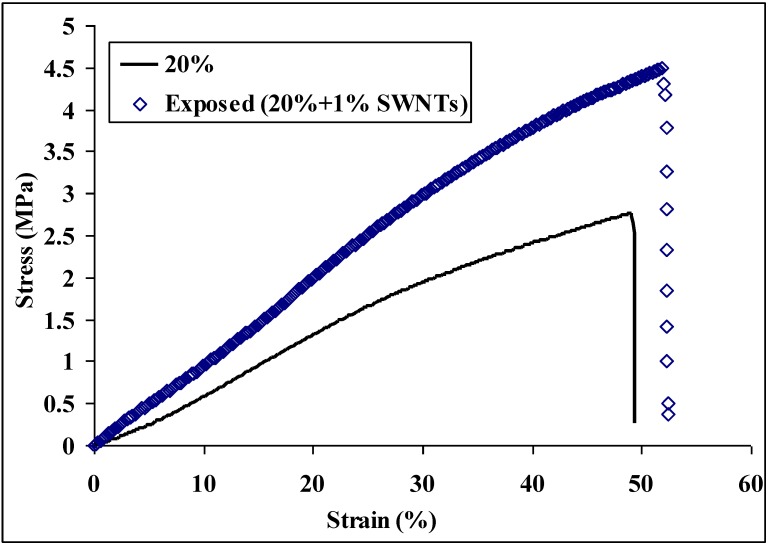
Typical stress-strain plots of nylon 6 non-woven fiber mats electrospun from 20 wt %, and 20 wt % with 1 wt % CNTs after formic acid vapor exposure for 30 min at room temperature.

**Figure 8:**
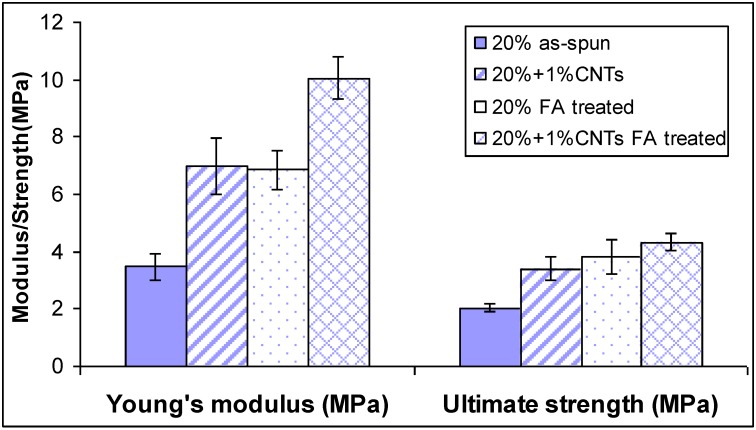
Mechanical properties of nylon 6 non-woven fiber mats electrospun from 20 wt % solutions and 20 wt % nylon 6 solutions with 1 wt % CNTs before/after formic acid vapor exposure for 30 min at room temperature.

We have proved that Young’s modulus, tensile strength, and toughness of the nylon 6 non-woven fiber mats increased 51%, 87%, and 136%, respectively, after incorporating 1 wt % CNTs into the nylon 6 nanofibers. This increase was attributed to increased fiber mechanical properties. After exposing the nylon 6 non-woven fiber mats (no CNTs) to formic acid vapor for 30 min at room temperature, Young’s modulus, tensile strength, and toughness of the nylon 6 non-woven fiber mats increased 56%, 67%, and 39%, respectively, based on increased fiber-fiber bonding. Figure 7 shows the typical stress-strain plots of nylon 6 non-woven fiber mats electrospun from 20 wt % solutions and non-woven fiber mats electrospun from 20 wt % with 1 wt % CNTs after vapor exposure. The tensile properties are summarized in Table 4. Figure 8 shows the comparison of the mechanical properties of these two non-woven fiber mats. Young’s modulus of the nylon 6 non-woven fiber mats electrospun from 20 wt % with 1 wt % CNTs after vapor exposure was increased 106%, which is the addition of the increase by incorporating CNTs into nylon 6 nanofibers and by vapor exposure. The improvement of mechanical properties of nylon 6 is significant.

### 2.3. Increase Packing Density of Electrospun Nylon 6 Non-Woven Fiber Mats to Increase Mechanical Properties

**Figure 9:**
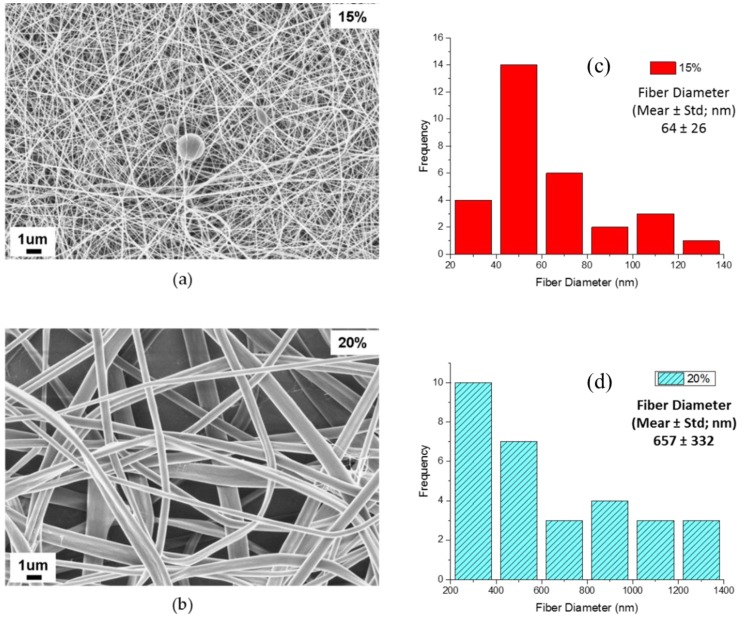
Field emission scanning electron microscope (FESEM) imaging of nylon 6 fibers electrospun from: (**a**) 15 wt % nylon 6 in 88% formic acid solutions; (**b**) 20 wt % nylon 6 in 88% formic acid solutions; (**c**) fiber diameter distribution from FESEM image (**a**) of nylon 6 electrospun from 15 wt % solutions; and (**d**) fiber diameter distribution from FESEM image (**b**) of nylon 6 electrospun from 20 wt % solutions.

A final approach for increasing the 2-D-structured electrospun nylon 6 non-woven fiber mat’s strength was to increase the packing density of fibers by decreasing the fiber diameter. Figure 9 shows the morphology of nylon 6 fibers electrospun from 15 wt % (Figure 9a) and 20 wt % (Figure 9b) nylon 6 in 88% formic acid. Thirty measurements were conducted to analyze the average fiber diameter for using software Image J. The average diameter of nylon 6 fibers electrospun from 20 wt % and 15 wt % solutions is 657 ± 332 nm (Figure 9d) and 64 ± 26 nm (Figure 9c), respectively. Thin but beaded fibers were formed from the 15 wt % solution. The average fiber diameter of the nylon 6 nanofiber electrospun from 20 wt % solutions is much bigger than the nylon 6 fibers electrospun from a 15 wt % solution. Higher concentration of nylon 6 favored the formation of uniform fibers.

**Figure 10:**
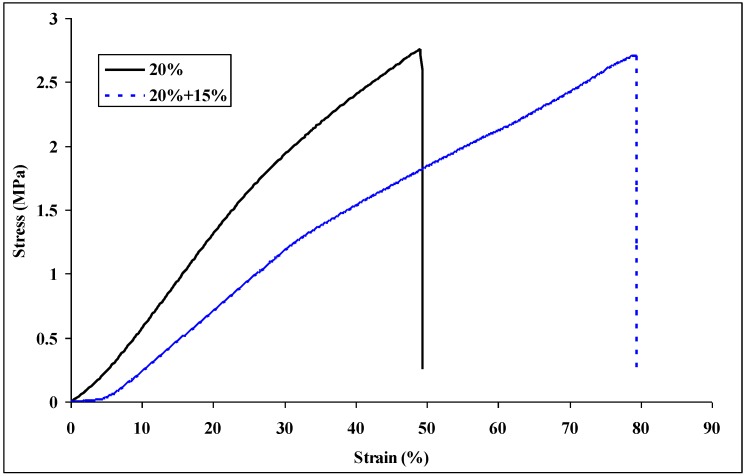
Typical stress-strain plots for the nylon 6 non-woven fiber mats electrospun from 20 wt %, 20 wt % and 15 wt % nylon 6 in 88% formic acid solutions.

To fully realize the theoretically predicted performance improvement, a number of important factors must be manifested in the final composite [10]. For a structural point of view, the fiber spacing should be smaller than the characteristic strength-limiting flaw; thus, smaller fiber diameters are desirable in order to produce a higher packing density. Figure 10 shows the typical stress-strain plots of nylon 6 non-woven fiber mats electrospun from 20 wt % and 20 wt % + 15 wt % solutions. Young’s modulus, tensile strength, elongation, and toughness of the non-woven fiber mats are summarized in Table 5. The elongation and toughness of the nylon 6 non-woven fiber mats electrospun from 20 wt % and 15 wt % solutions increased 39% and 75%, respectively, compared to the non-woven fiber mats electrospun from 20 wt %. The smaller size diameter of the nylon 6 nanofibers electrospun from 15 wt % solutions caused a higher packing density and hence improved the ductility of the electrospun nylon 6 non-woven fiber mats. Papkov *et al.* [13] has demonstrated improvements in modulus and strength of individual electrospun polymer nanofibers with a reduction in their fiber diameter. This size effect of nanofiber diameter to the mechanical properties has also been proved in this study. The non-woven fiber mats electrospun from 20 wt % and 15 wt % of solutions showed higher mechanical properties than those from 20 wt % solutions.

## 3. Materials and Methods

### 3.1. Materials

Nylon 6 (*M*_w_ = ~10,000 Da) and 88% formic acid were purchased from Sigma-Aldrich (St. Louis, MO, USA) and VWR International (Radnor, PA, USA), respectively. Single-walled Carbon Nanotubes (CNTs) was obtained from Cheap Tubes Inc. (Cambridgeport, VT, USA). All materials were used as received.

### 3.2. Preparation of Electrospun Solutions

Nylon 6 was dissolved in 88% formic acid at concentrations of 10 wt %, 15 wt %, and 20 wt % with a wrist-action shaker (Burrell Scientific Inc., Pittsburgh, PA, USA) for 24 h prior to electrospinning. One percent (based on the weight of nylon 6) of CNTs was added to the 15 wt % and 20 wt % nylon 6/formic acid solutions and mixed with the wrist-action shaker for 24 h before electrospinning.

### 3.3. Electrospinning

**Figure 11:**
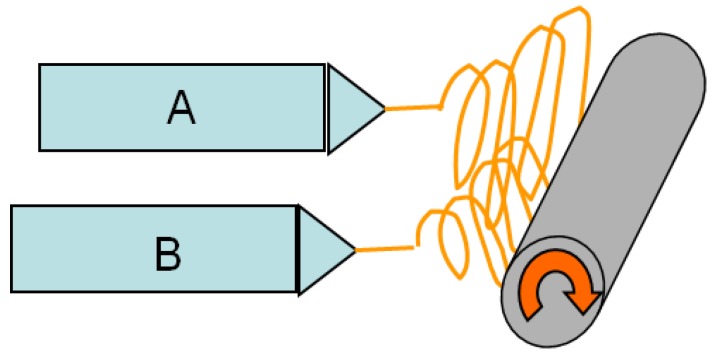
Schematic for horizontal paralleled electrospinning setup.

A horizontal paralleled (two syringes working at the same time) electrospinning setup (Figure 11) was used in this work. During electrospinning, the nylon 6 solutions were introduced into two 5-mL plastic syringes (VWR Scientific, West Chester, PA, USA). Each syringe was attached to a 20-gauge needle (Hamilton 90020, VWR Inc.) collected to a high voltage supply (Gamma Model ES30, Ormond Beach, FL, USA), with a 25 kV voltage. The needle-to-collector distance was 10 cm. The applied electrical difference was 25,000 volts. The solution was fed at a rate of 0.5 mL/h using a syringe pump (Harvard apparatus pump 33, Holliston, MA, USA). The grounded collector was a rotating roll covered with aluminum foil. The samples were collected for three hours.

Table 6 shows the concentrations of the nylon 6 solutions used for the parallel electrospinning (Figure 11). For the non-woven fiber mats electrospun from 20 wt % and 15 wt %, 20 wt % and 10 wt %, 15 wt % and 10 wt % solutions, the syringes A and B shown in Figure 11 were switched at the halfway point of the 3-h electrospinning.

### 3.4. Post Treatment of Electrospun Nylon 6 Non-Woven Fiber Mats

Thermal bonding of the electrospun nylon 6 non-woven fiber mats was achieved by free anneal at 70 °C for 24 h. To achieve solvent bonding, the electrospun nylon 6 non-woven fiber mats were laid on top of a fiberglass mesh (20 × 20 mesh), which covered a 250-mL beaker containing 100 mL formic acid for 30 min at room temperature.

### 3.5. Scanning Electron Microscopy

Examination of the morphology and fiber diameters of the electrospun nylon 6 non-woven fiber mats was done using a Leica 440 scanning electron microscope (SEM, Leica Microsystems Inc., Buffalo Grove, IL, USA) at 30 kV, and a LEO 1550 Field Emission Scanning Electron Microscope (FESEM, Carl Zeiss AG, Oberkochen, Germany) at a voltage of 3 kV, using an in-lens detector. Samples were coated for 30 s with 10 nm Au-Pd to prevent charging.

### 3.6. Mechanical Testing

The mechanical testing was conducted according to ASTM standard D638-14 [23] with an Instron 5566 (INSTRON, Boston, MA, USA) equipped with a 100-N load-cell at 65% RH and 23 °C. The test measures the Young’s modulus, tensile stress at break, and tensile strain at break that the electrospun nylon 6 non-woven fiber mats can survive prior to failure. The thickness of the nylon 6 non-woven fiber mats was measured with a micrometer. The crosshead speed was 10 mm/min. The electrospun nylon 6 non-woven fiber mats were punched into dog-bone specimens with a dimension of 63.5 × 9.53 mm^2^ (Die ASTM D-638 type V [23], ODC Tooling & Molds, Batavia, IL, USA) using a manual test specimen cutting clicker press (Lucris MA Series 3, Toronto, ON, Canada).

## 4. Conclusions

Young’s modulus, tensile strength, and toughness of the nylon 6 non-woven fiber mats electrospun from 20 wt % solutions increased 51%, 87%, and 136%, respectively, after incorporating 1 wt % CNTs into the nylon 6 nanofibers. With the addition of beaded nylon 6 nanofibers into the non-woven fiber mats, the nylon 6 non-woven fiber mats electrospun from 20 wt % and 10 wt % demonstrated a statistically significant increase in Young’s modulus when compared with nylon 6 non-woven fiber mats electrospun from 20 wt %. After annealing, tensile strength, elongation and toughness of the nylon 6 non-woven fiber mats electrospun from 20 wt % and 10 wt % solutions increased 26%, 28%, and 68% compared to those from 20 wt % solutions. Young’s modulus, tensile strength, and toughness of the nylon 6 non-woven fiber mats increased 56%, 67%, and 39%, respectively, after formic acid vapor exposure for 30 min at room temperature. The smaller size diameter of the nylon 6 nanofibers electrospun from 15 wt % solutions caused a higher packing density, and hence improved the ductility of the electrospun nylon 6 non-woven fiber mats.

## Figures and Tables

**Table 1:** Tensile properties of nylon 6 non-woven fiber mats electrospun from 20 wt % nylon 6 with/without CNTs.

Tensile Properties	20%	20% + 1% CNTs
Young‘s modulus (MPa)	4.81 ± 1.03	7.25 ± 1.96
Tensile strength (MPa)	2.03 ± 0.99	3.80 ± 1.87
Elongation (%)	54.1 ± 14.6	63.7 ± 19.2
Toughness (MPa)	0.64 ± 0.48	1.51 ± 1.05

**Table 2:** Tensile properties of nylon 6 non-woven fiber mats electrospun from 20 wt % and 10 wt % solutions before and after annealing at 70 °C for 24 h.

Tensile Properties	Before Annealing	After Annealing
Young’s modulus (MPa)	4.81 ± 1.03	4.12 ± 1.33
Tensile strength (MPa)	1.63 ± 0.50	2.06 ± 0.72
Elongation (%)	45.6 ± 13.2	58.2 ± 11.4
Toughness (MPa)	0.41 ± 0.23	0.69 ± 0.30

**Table 3:** Tensile properties of nylon 6 non-woven fiber mats electrospun from 20 wt % solutions before and after formic acid vapor exposure for 30 min at room temperature.

Tensile Properties	Before Exposure	After Exposure
Young’s modulus (MPa)	4.81 ± 1.03	7.50 ± 2.41
Tensile strength (MPa)	2.03 ± 0.99	3.40 ± 1.26
Elongation (%)	54.1 ± 14.6	48.1 ± 8.9
Toughness (MPa)	0.64 ± 0.48	0.89 ± 0.55

**Table 4:** Tensile properties of nylon 6 non-woven fiber mats electrospun from 20 wt % solutions, and from 20 wt % nylon 6 solution with 1 wt % CNTs after formic acid vapor exposure for 30 min at room temperature.

Tensile Properties	20%	Exposed (20% + 1% CNTs)
Young’s modulus (MPa)	4.81 ± 1.03	9.90 ± 2.20
Tensile strength (MPa)	2.03 ± 0.99	4.33 ± 0.94
Elongation (%)	54.1 ± 14.6	50.0 ± 4.7
Toughness (MPa)	0.64 ± 0.48	1.25 ± 0.36

**Table 5:** Tensile properties of nylon 6 non-woven fiber mats electrospun from 20 wt %, 20 wt %, and 15 wt % solutions.

Tensile Properties	20%	20% + 15%
Young’s modulus (MPa)	4.81 ± 1.72	4.48 ± 1.65
Tensile strength (MPa)	2.03 ± 0.99	2.59 ± 1.25
Elongation (%)	54.1 ± 14.6	75.4 ± 12.2
Toughness (MPa)	0.64 ± 0.48	1.12 ± 0.77

**Table 6:** The concentrations of the nylon 6 solutions used for parallel electrospinning.

A	B	Feed Rate (mL/h)
20%	20%	0.5
20%	15%	0.5
20%	10%	0.5
15%	15%	0.5
15%	10%	0.5
